# Therapeutic potential of the gut commensal bacterium *Parabacteroides goldsteinii* in human health and disease treatment

**DOI:** 10.3389/fmicb.2025.1618892

**Published:** 2025-09-11

**Authors:** Ziyun Li, Li Zhang, Zhenxia Wan, Huijuan Liu, Ting Zhang, Yan Li

**Affiliations:** Shandong Provincial Maternal and Child Health Care Hospital Affiliated to Qingdao University, Jinan, China

**Keywords:** *Parabacteroides goldsteinii*, immune regulation, metabolic regulation, disease association, therapeutic potential

## Abstract

The gut microbiota, as a critical guardian of human health, maintains physiological homeostasis, modulating immunity, and facilitates nutrient metabolism. *Parabacteroides goldsteinii*, a probiotic gut commensal, has garnered increasing scientific attention. This review systematically examines its biological characteristics, then analyzes mechanisms promoting health (immunomodulation, metabolic regulation, and intestinal barrier reinforcement), and finally evaluates disease associations (metabolic disorders, neurological diseases, inflammatory conditions, and malignancies). Current evidence shows that therapeutic efficacy against obesity, non-alcoholic fatty liver disease, inflammatory bowel disease, autism spectrum disorder, and colorectal cancer via short-chain fatty acids secretion, bile acid transformation, and host immunity modulation. Dietary factors (e.g., inulin), pharmacological agents (e.g., metformin, aspirin), and lifestyle interventions (e.g., exercise synbiotics) dynamically regulate its abundance, underscoring therapeutic potential. Despite translational challenges–like optimizing cultivation, dose-response characterization, and genetic tool development–emerging applications (engineered probiotics, fecal microbiota transplantation, and synthetic biology) highlight broad prospects. Future research should prioritize context-dependent mechanisms across diseases and refined translation strategies for microbiome-based precision medicine.

## Graphical Abstract

Therapeutic potential of *Parabacteroides goldsteinii*. Created in BioRender. Dwad, D. (2025) https://BioRender.com/fg89trz. 
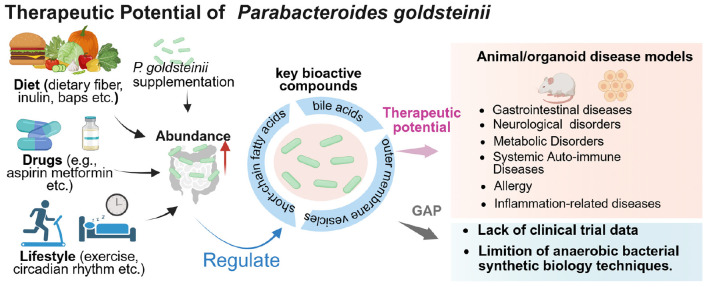

## 1 Introduction

The gut microbiota serves as a pivotal health guardian, with its intricate ecosystem playing indispensable roles in maintaining physiological homeostasis, modulating immune responses, facilitating nutrient metabolism, and preventing diseases. In recent years, rapid advancements in multi-omics technologies, such as metagenomics and metabolomics, have enabled scientists to delve deeper into the complexities of gut microbial communities and unravel the profound connections between specific microbial species and host health.

Among these, *Parabacteroides goldsteinii*–a prevalent commensal bacterium in the human gastrointestinal tract–has emerged as a research focus due to its close associations with host immunomodulation, metabolic homeostasis, and disease pathogenesis. Elucidating the biological characteristics, colonization dynamics, mechanistic actions, and disease correlations of *P. goldsteinii* not only advances our understanding of the molecular underpinnings of host-microbiota interactions but may also provide a foundation for exploring microbiota-based strategies in disease prevention and therapeutic innovation. However, a systematic synthesis of *P. goldsteinii*’s multifaceted mechanisms across disease contexts–and its translational challenges–remains limited. This review systematically examines first its biological characteristics and colonization dynamics, then evaluates its mechanistic roles in immune/metabolic regulation and disease pathogenesis (spanning metabolic, neurological, inflammatory disorders, and oncology), and finally discusses translational applications [probiotics, fecal microbiota transplantation (FMT), synthetic biology] and unresolved challenges. By consolidating dispersed evidence and identifying clinical translation barriers, we aim to establish *P. goldsteinii* as a keystone modulator in microbiome-targeted therapies and accelerate its therapeutic deployment.

## 2 Comprehensive characteristics of identified *P. goldsteinii*

*Parabacteroides goldsteinii*, originally isolated from the feces of healthy adults and formerly classified as *Bacteroides goldsteinii* (Song et al., 2005; Sakamoto and Benno, 2006), is a strictly anaerobic, Gram-negative, rod-shaped obligate anaerobe belonging to the phylum Bacteroidetes, class Bacteroidia, order Bacteroidales, and genus *Parabacteroides*. This bacterium exhibits a negative indole test and forms circular colonies (1–2 mm in diameter) on Columbia blood agar medium, characterized by smooth margins, opaque grayish-white coloration, central elevation, smooth surface texture, and moist consistency (Sakamoto and Benno, 2006). Colonies become transferable within 3–4 days of incubation under optimal growth conditions at 37 °C, reflecting its adaptation to colonize the low-oxygen microenvironment of the human gastrointestinal tract, where it predominantly resides in both healthy individuals and subsets of patients with gastrointestinal disorders.

*Parabacteroides goldsteinii* primarily generates acetate and succinate via glucose metabolism. These fermentation products serve as critical short-chain fatty acids (SCFAs), acetate directly, and succinate indirectly via conversion by other microbes, contribute to intestinal health maintenance and host metabolic regulation (Cui et al., 2022). Furthermore, this bacterium modulates BA transformation, thereby influencing gut microbial equilibrium, mucosal barrier integrity, and host immune responses (Li et al., 2024b,^2025^).

## 3 Regulatory mechanisms of *P. goldsteinii* in human health

**FIGURE 1:**
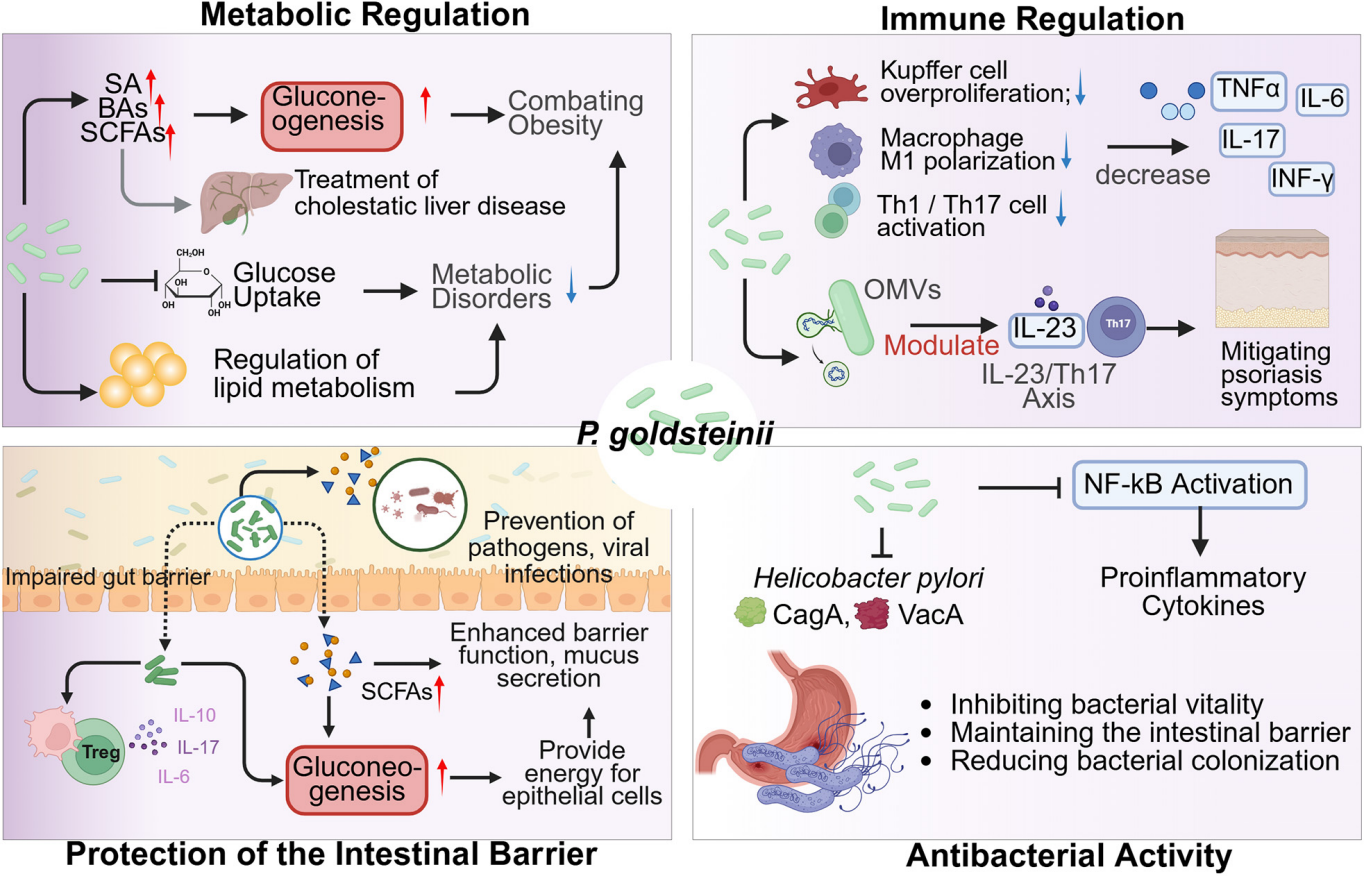
Mechanisms of *P. goldsteinii* in human health. SA, succinic acid. A red upward arrow denotes an increase, while a blue downward arrow indicates a decrease. A straight line at the arrow’s tip signifies inhibition. Created in BioRender. Dwad, D. (2025) https://BioRender.com/xbd2c2n.

*Parabacteroides goldsteinii*, as an essential component of the human gut microbiota, serves as a multifaceted modulator of host health through immune-metabolic crosstalk (Figure 1). *P. goldsteinii* modulates the host immune system to influence the pathogenesis and progression of autoimmune diseases (Chang et al., 2024; Ye et al., 2025). *P. goldsteinii* mitigates autoimmune pathogenesis by suppressing macrophage M1 polarization, aberrant hyperproliferation of Kupffer cells (KCs), and activation of Th1 or Th17 lymphocytes, thereby inhibiting the release of proinflammatory cytokines the release of pro-inflammatory cytokines, including tumor necrosis factor-α (TNF-α), interleukin-6 (IL-6), interleukin-17 (IL-17), and interferon-γ (IFN-γ) (Figure 1). Notably, it significantly reduces imiquimod-induced systemic lupus erythematosus (SLE) in murine models by reducing immune-inflammatory responses (Chang et al., 2024). The outer membrane vesicles (OMVs) of *P. goldsteinii* translocate to arthritic joints, where they activate the Cav-1–Nrf2 axis to suppress neutrophil extracellular trap (NET) formation, thereby alleviating rheumatoid arthritis (RA) severity (Ye et al., 2025). Oral administration of *P. goldsteinii*-derived OMVs (*Pg*-OMVs) enables their colonic permeation, systemic circulation, and subsequent accumulation in psoriatic skin lesions. This process reduces epidermal hyperplasia and inflammatory leukocyte infiltration via downregulating IL-23/Th17 signaling (Su et al., 2025). Furthermore, *P. goldsteinii* ameliorates colitis by inhibiting lipopolysaccharide (LPS)-mediated activation of the PI3K-Akt pathway in colonic macrophages (Tsou et al., 2021; Li et al., 2024c). Intriguingly, its abundance negatively correlates with tumor metastasis, suggesting potential antitumorigenic properties (Yuan et al., 2022b).

*Parabacteroides goldsteinii* enhances host intestinal gluconeogenesis and barrier integrity by promoting succinate and secondary BA biosynthesis, which ameliorates metabolic disorders such as cholestatic liver disease (Zhang et al., 2023; Li et al., 2025). Its regulation of lipid metabolism-through modulation of hepatic farnesoid X receptor (FXR) and adipose takeda G protein-coupled receptor 5 (TGR5) signaling-confers protective effects against atherosclerosis and cerebrovascular dysfunction (Wu et al., 2022). The SCFAs (e.g., acetate, butyrate) secreted by *P. goldsteinii* serve as both energy substrates for colonic epithelial cells and activators of histone deacetylase 3-dependent tight junction protein synthesis, thereby fortifying the mucosal barrier against pathogen translocation (Wu et al., 2019). In summary, *P. goldsteinii* orchestrates systemic health benefits through immunomodulatory precision, metabolic fine-tuning, barrier reinforcement, and microbiota-driven pathogen exclusion, positioning it as a promising therapeutic target for inflammatory and metabolic pathologies (Figure 1).

## 4 *P. goldsteinii*: a microbial key player in disease regulation

**FIGURE 2:**
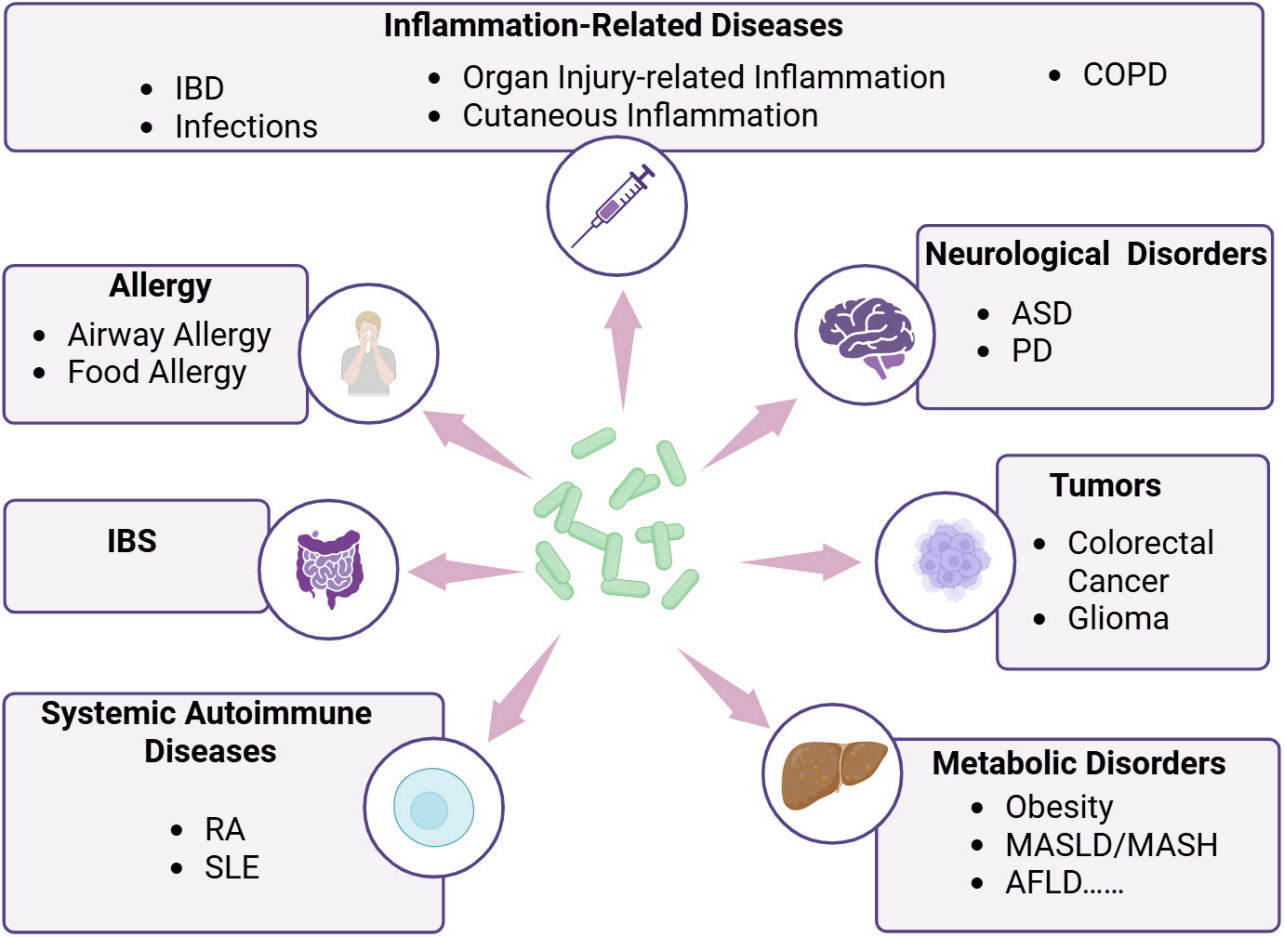
*Parabacteroides goldsteinii* has regulatory effects on a variety of diseases. Created in BioRender. Dwad, D. (2025) https://BioRender.com/rll9yr6.

*Parabacteroides goldsteinii* is recognized as a gut commensal bacterium exhibiting probiotic potential rather than pathogenic traits. This bacterium indicates therapeutic benefits in ameliorating diverse chronic inflammation-related diseases (Figure 2). Specifically, its strain RV-01 exerts anti-inflammatory effects and potential probiotic characteristics, qualifying as a safe functional food ingredient (Lin et al., 2024). Although *P. goldsteinii sp.* nov. was initially isolated from human blood in 2009 (Sakamoto et al., 2009) and subsequently from the blood of peritonitis patients in 2018 (Kim et al., 2018), no conclusive evidence suggests its direct pathogenicity. Conversely, this species has shown disease-alleviating effects across multiple disorders, including metabolic disorders such as obesity, metabolic-associated fatty liver disease (MASLD), metabolic-associated steatohepatitis (MASH), type 1 diabetes (T1D), and alcoholic fatty liver disease (AFLD); neuroregulatory disorders such as autism spectrum disorder (ASD) and Parkinson’s disease (PD); tumors; allergies; irritable bowel syndrome (IBS); systemic autoimmune diseases; and inflammatory-related diseases such as inflammatory bowel disease (IBD) and chronic obstructive pulmonary disease (COPD) (Figure 2 and Table 1).

**TABLE 1:** *Parabacteroides goldsteinii* as a therapeutic agent: target diseases, observed effects, and proposed mechanisms.

Strain	Disease (model)	Mechanisms	Reference
JCM 13446	Obesity (high-fat diet (HFD)-induced obese mouse)	Live *P. goldsteinii* increased thermogenesis in adipose tissue, enhanced gut integrity, and reduced levels of inflammation and insulin resistance.	Wu et al., 2019
MTS01	ASD (mouse model of ASD-like offspring)	In the gut, it enhanced neuropeptide signaling and suppressed aberrant proliferation/inflammation; in the hippocampus, it upregulated ribosomal-mitochondrial functions and antioxidant defenses while downregulating glutamatergic transmission.	Lin et al., 2022
MTS01	Gastritis caused by *Helicobacter pylori infection* (mouse)	Altering the gut microbiota composition in mice significantly reduced serum cholesterol levels and mitigated the pathogenic effects of *H. pylori* VacA and CagA on gastric epithelial cells.	Lai et al., 2022a
MTS01	COPD (mouse model with tobacco exposure)	LPS derived from *P. goldsteinii* exerts anti-inflammatory effects and significantly ameliorates COPD by acting as an antagonist of the toll-like receptor 4 (TLR4) signaling pathway.	Lai et al., 2022b
/	Anastomotic leakage (AL) in colorectal cancer (CRC) (CRC mouse model)	Modulate mucosal pro-inflammatory cytokines, reduce the expression of MIP-1α, MIP-2, MCP-1, and IL-17A/F, thereby alleviating inflammatory responses and promoting the healing process.	Hajjar et al., 2023
JCM 13446	Aspirin-mediated intestinal injury (mouse)	Supplementation of *P. goldsteinii* or its metabolite 7-keto-lithocholic acid (7-keto-LCA) promotes intestinal epithelial repair by inhibiting the signaling of the intestinal BA receptor FXR	Li et al., 2024b
RV-01	HCECs and healthy mice.	The autoclaved *P. goldsteinii* RV-01 retains its anti-inflammatory effects in human colonic epithelial cells (HCECs), and animal toxicity studies have yielded negative results.	Lin et al., 2024
JCM 13446	SLE (mouse)	*P. goldsteinii* reduces spleen weight, proteinuria, and the increase in serum anti-DNA autoantibodies and STAT4 levels. Additionally, it improves renal and hepatic function markers, such as creatinine, blood urea nitrogen, glomerular injury, fibrosis, and serum liver enzymes.	Chang et al., 2024
/	Aging-related infections (mouse)	Live *P. goldsteinii* colonization prevents age-related infections via apigenin-mediated antagonism of Fgr (M341/D404), which rescues Vav1 phosphorylation to trigger Cdc42/Rac1-Arp2/3 signaling and actin-dependent phagocytosis activation in macrophages, restoring bacterial clearance in aged hosts.	Gu et al., 2025
GDMCC1.2815	RA (collagen-induced arthritis mouse)	The *P. goldsteinii* enriched in Guizhi Shaoyao Zhimu Decoction (GSZD) secretes OMVs that migrate to the joints, activating the Cav-1-Nrf2 axis, thereby reducing the formation of NETs and alleviating arthritis.	Ye et al., 2025
JCM 13446	Psoriasis (psoriasis-like mouse)	After the *Pg*- OMVs cross the intestinal barrier and circulate to the inflamed skin of psoriasis-like mice, they reduce epidermal hyperplasia, inhibit the infiltration of inflammatory cells into skin lesions, and effectively improve both skin and systemic inflammation.	Su et al., 2025

### 4.1 Metabolic disorders

**FIGURE 3:**
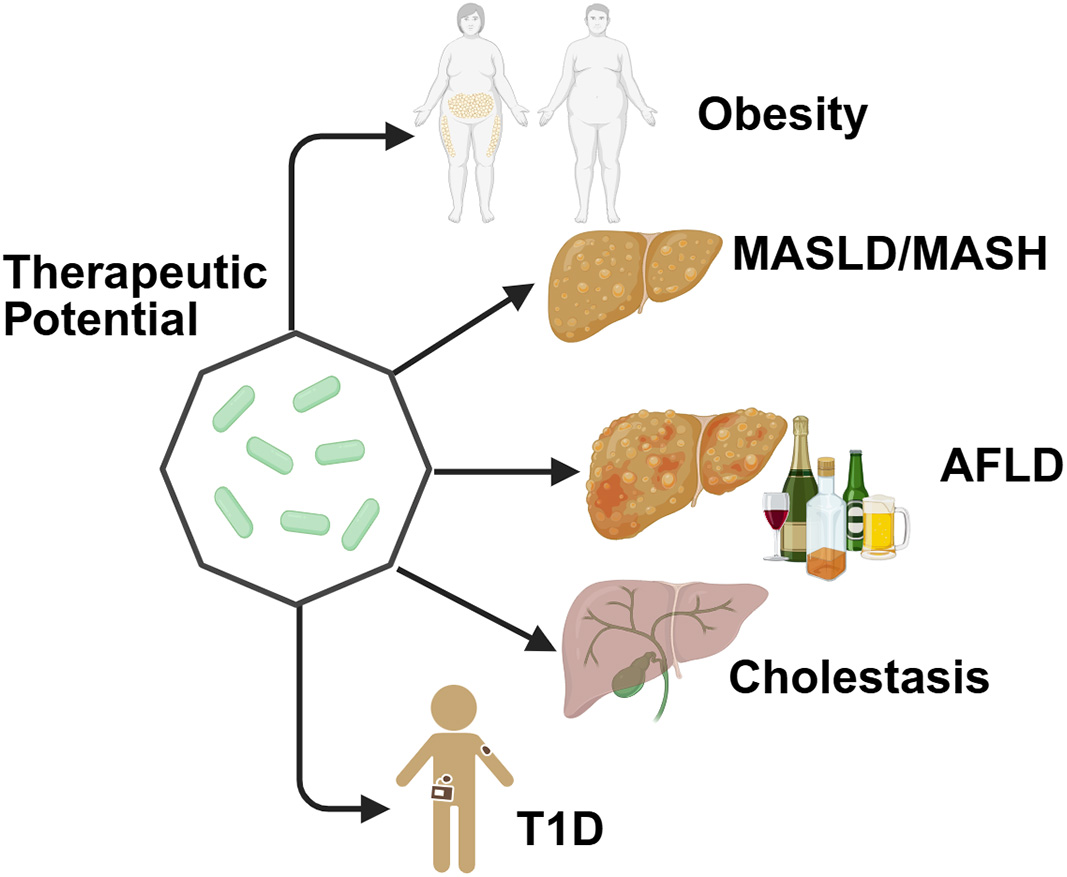
Therapeutic benefits of as a potential probiotic for multiple metabolic diseases. Created in BioRender. Dwad, D. (2025) https://BioRender.com/93cnail.

*Parabacteroides goldsteinii* emerges as a central regulator of metabolic homeostasis across diverse pathological conditions, including obesity, MASLD, AFLD, and immune-mediated metabolic disorders (Figure 3). *P. goldsteinii* can upregulate the concentration of 7-Keto-LCA in the gut, which has been proven to be an FXR antagonist that promotes Wnt signaling, thereby facilitating the self-renewal of intestinal stem cells (Li et al., 2024b). Its therapeutic effects are mediated through remodeling of gut microbiota, regulation of bioactive metabolites (e.g., SCFAs, BAs), reinforcement of intestinal barrier function, and suppression of inflammatory cascades, positioning it as a promising probiotic candidate for microbiome-targeted interventions.

#### 4.1.1 Obesity

*Parabacteroides goldsteinii* ameliorates HFD-induced obesity, inflammation, and insulin resistance by modulating gut microbiota composition, remodeling the microbial-derived metabolic landscape (e.g., SCFAs and BAs), and enhancing intestinal barrier function. Animal-level studies have shown that its abundance correlates positively with prebiotics intake [e.g., propolis, eggshell membrane, chitosan oligosaccharides (COS), and polyethylene glycol] (Chang et al., 2015, 2019; Wu et al., 2019), suggesting its probiotic potential against obesity-associated metabolic disorders. For instance, the >300 kDa polysaccharide fraction H1 selectively enriches *P. goldsteinii*, leading to attenuated HFD-induced weight gain and metabolic dysregulation in mice (Wu et al., 2019). Direct administration of live *P. goldsteinii* also reduces adiposity, stimulates adiposity thermogenesis (as evidenced by significantly increased expression of thermogenic markers including uncoupling protein 1 and PR domain-containing 16), restores gut integrity, and alleviates systemic inflammation and insulin resistance (Wu et al., 2019).

Mechanistically, specific dietary interventions exhibit *P. goldsteinii*-dependent therapeutic effects: (i) Propolis ethanol extract and eggshell membrane reduce HFD-driven obesity and insulin resistance via microbial community restructuring and *P. goldsteinii* enrichment (Cai et al., 2020; Ramli et al., 2020); (ii) COS inhibit HFD-induced weight gain and metabolic dysfunction by increasing *P. goldsteinii* levels (Wang et al., 2021); (iii) Prebiotic blend restore antibiotic-disrupted gut microbiota dysbiosis by selectively enhancing *P. goldsteinii* colonization (Yuan et al., 2022a); (iv) 40% polyethylene glycol (PEG400) reduces adiposity and adipose inflammation through *P. goldsteinii*-dependent mechanisms (Ishibashi et al., 2023).

#### 4.1.2 Metabolic associated fatty liver disease (MASLD)

Emerging evidence underscores the gut microbiota’s pivotal role in MASLD, particularly its inflammatory subtype (MASH), where *P. goldsteinii* emerges as a critical regulator (Ishioka et al., 2017; Lange et al., 2024). Dietary patterns profoundly influence gut microbial composition and functionality, which in turn regulate hepatic inflammation and metabolism. In multiple diet-induced murine models, the severity of steatohepatitis correlates closely with gut microbiota alterations. Notably, choline-deficient L-amino acid-defined (CDAA) diets markedly reduce the abundance of the anti-inflammatory bacterium *P. goldsteinii*, which is strongly associated with elevated IL-17 levels in the ileum and portal vein, alongside upregulated hepatic chemokine expression (Ishioka et al., 2017). These findings suggest that gut dysbiosis may exacerbate liver inflammation through proinflammatory mechanisms.

In nutritional models of MASLD, L-ornithine L-aspartate (LOLA) treatment significantly enriches *P. goldsteinii* and modulates metabolic pathways (e.g., energy metabolism, nucleotide biosynthesis), suggesting its therapeutic potential via microbial metabolic optimization (Lange et al., 2024). Urolithin C (UroC) ameliorates choline-deficient high-fat diet (CDAHFD)-induced MASLD mice by: (i) enriching *P. goldsteinii* colonization, (ii) strengthening the intestinal mucosal barrier, and (iii) activating hepatic AMP-activated protein kinase (AMPK)–collectively reducing liver injury and metabolic disturbances (Xu et al., 2023).

Collectively, *P. goldsteinii* plays a pivotal role in MASLD pathogenesis: depletion aggravates gut-liver inflammation, whereas targeted replenishment (pharmacological/nutraceutical) improves outcomes. Future work must dissect its mechanistic roles in gut barrier maintenance, immune modulation, and metabolic crosstalk.

#### 4.1.3 Alcoholic fatty liver disease (AFLD)

The gut microbiota plays a pivotal role in AFLD, and modulation of its composition and functionality represents a promising therapeutic strategy to alleviate alcohol-induced hepatic injury (Malaguarnera et al., 2014; Shen et al., 2024). In murine models of chronic alcohol consumption, rhubarb extract supplementation restructured gut microbial communities, particularly enhancing *P. goldsteinii* proliferation, which significantly correlated with attenuated alcohol-driven hepatic inflammation and oxidative stress (Neyrinck et al., 2017). These findings suggest that *P. goldsteinii* enrichment may protect against AFLD by restoring microbial equilibrium, suppressing proinflammatory cascades, and reducing oxidative damage.

Mechanistically, *P. goldsteinii* enrichment is associated with: (i) Gut ecosystem optimization–improved microbial diversity and metabolic output; (ii) Intestinal barrier reinforcement–enhanced tight junction integrity and reduced endotoxin translocation; (iii) Hepatic cytoprotection–reduced proinflammatory cytokine (e.g., TNF-α, IL-6) and oxidative stress markers (e.g., malondialdehyde) (Neyrinck et al., 2017). These findings establish a scientific foundation for microbiota-targeted AFLD interventions and identify *P. goldsteinii* as a potential therapeutic target.

#### 4.1.4 Other metabolic disorders

Emerging studies indicate that the traditional Chinese herbal formula Si-Ni-San (SNS) significantly ameliorates partial bile duct ligation (pBDL)-induced cholestatic liver injury by modulating gut microbiota and enhancing intestinal barrier function (Li et al., 2025). The therapeutic efficacy is primarily mediated by selective *P. goldsteinii* enrichment, suggesting novel therapeutic strategies for cholestatic liver diseases and highlighting this bacterium’s probiotic potential. Anti-PD-1 immunotherapy accelerates T1D onset in non-obese diabetic (NOD) mice but also alters gut microbial diversity and composition (Patel et al., 2023). *P. goldsteinii* emerged as a potential “rescue” bacterium, contrasting sharply with insulin resistance-associated microbes such as *Akkermansia muciniphila*. This dichotomy suggests targeted microbial modulation could counteract immunotherapy-associated adverse events, supporting microbiome-based adjuvant therapies.

### 4.2 Neurological disorders

*Parabacteroides goldsteinii* functions as a putative beneficial bacterium, exerting therapeutic effects through gut microbiota modulation, intestinal barrier reinforcement, and subsequent attenuation of inflammatory responses and neurotransmitter balance restoration. Its bioactive components (e.g., LPS and zwitterionic polysaccharides) maintain anti-inflammatory activity post-inactivation (Lin et al., 2024). The link between neuropathology and chronic low-grade inflammation suggests *P. goldsteinii* could ameliorate symptoms through dual mechanisms: microbiota regulation and systemic inflammation reduction (Lin et al., 2022; Morel et al., 2023; Wei et al., 2024; Zeng et al., 2024).

#### 4.2.1 Autism spectrum disorder (ASD)

Recent studies have identified *P. goldsteinii* as a potential therapeutic agent in ASD models (Lin et al., 2022; Morel et al., 2023). In maternal immune activation (MIA)-induced ASD murine models, administration of *P. goldsteinii* MTS01 significantly reduced intestinal and systemic inflammation while improving ASD-associated behavioral deficits–effects mediated through modulation of neuropeptide signaling pathways and enhanced antioxidant activity (Lin et al., 2022). Moreover, perinatal exposure to broad-spectrum antibiotics (e.g., ampicillin) disrupted maternal gut microbiota, leading to ASD-related behavioral phenotypes in offspring, characterized by reduced social motivation, increased anxiety-like behaviors, and altered ultrasonic vocalization patterns. These behavioral abnormalities correlated with reduced oxytocin receptor expression in the prefrontal cortex and gut microbiota dysbiosis, particularly significant reductions in *P. goldsteinii* abundance (Morel et al., 2023).

#### 4.2.2 Parkinson’s disease (PD)

Emerging evidence has linked dietary patterns to PD risk, with recent studies highlighting a significant inverse association between PD incidence and the consumption of a food component designated as “baps, the soft white bread rolls”–an effect potentially mediated by gut microbiota modulation, particularly increased *P. goldsteinii* abundance (Zeng et al., 2024). Furthermore, a co-fermentation product of black barley and quinoa with lactic acid bacteria (designated FG) was shown to ameliorate HFD-induced cognitive impairment in murine models via multiple mechanisms: modulating gut dysbiosis (increasing *P. goldsteinii*–an anti-inflammatory bacterium–while reducing pro-inflammatory bacterial taxa), decreasing LPS production, and enhancing both intestinal and blood-brain barrier integrity (Wei et al., 2024). Together, these alterations suppress neuroinflammation and preserve neurotransmitter homeostasis. supporting further investigation into *P. goldsteinii*’s role in PD pathogenesis. It benefits may arise from anti-inflammatory effects, intestinal barrier reinforcement, and neuroprotection.

### 4.3 Inflammation-related diseases

Accumulating evidence in recent years has unveiled intricate connections between gut microbiota and various inflammation-associated disorders. Notably, *P. goldsteinii* has been identified as a putative probiotic agent, with documented ameliorative effects across multiple inflammatory conditions including but not limited to IBD, organ injury-related inflammation, cutaneous inflammation, infections, and COPD.

#### 4.3.1 Inflammatory bowel disease (IBD)

The pathogenesis of IBD involves complex interactions among genetic predisposition, environmental factors, and gut microbiota, with growing attention to specific microbial taxa. Emerging evidence highlights *P. goldsteinii* as a promising probiotic candidate with therapeutic potential in ameliorating intestinal inflammation and IBD symptoms. Clinical studies reveal that exclusive enteral nutrition (EEN) induces Crohn’s disease remission by reshaping gut microbial composition, while animal studies have confirmed that EEN significantly increases *P. goldsteinii* abundance and enhances intestinal barrier integrity (Jang et al., 2021). In Wiskott-Aldrich syndrome protein (WASP)-deficient murine models, *P. goldsteinii* abundance correlates inversely with fecal lipocalin-2 (LCN2), contrasting with the pro-inflammatory associations of *Helicobacter* and *Mucispirillum schaedleri*. This dichotomy underscores microbial balance in modulating intestinal inflammation (Tsou et al., 2021). Mechanistically, *P. goldsteinii* suppresses colitis by inhibiting the PI3K-Akt pathway, though LPS exposure abolishes this effect, suggesting functional dependence on immune crosstalk and ecological homeostasis within the gut microenvironment (Li et al., 2024c). Collectively, these findings reveal the multifaceted mechanisms through which *P. goldsteinii* may serve as a therapeutic target for IBD, supporting novel microbiome-directed treatment strategies.

#### 4.3.2 Organ injury-related inflammation

Emerging evidence indicates that *P. goldsteinii* and its metabolite 7-keto-LCA ameliorate aspirin-induced intestinal damage by inhibiting the FXR signaling pathway, promoting epithelial repair and intestinal stem cell regeneration. This mechanism highlights its role in gastrointestinal homeostasis (Li et al., 2024b). Additionally, the bacterium’s metabolic regulatory functions exhibit cross-organ protective effects: in antibiotic intervention models, probiotic supplementation (e.g., *Lactobacillus casei* Zhang) accelerates post-antibiotic recovery by enriching *P. goldsteinii*, elevating SCFA levels, and suppressing pro-inflammatory mediators such as IL-1α (Yao et al., 2021). Similarly, in acute kidney injury models, caloric restriction (CR) enhances *P. goldsteinii* and its metabolite dodecafluoropentane, simultaneously improving renal dysfunction, oxidative stress, and systemic inflammation (Zhu et al., 2024). These findings establish *P. goldsteinii* as a critical regulator within the microbiota-metabolite-host axis, orchestrating systemic homeostasis through multi-target mechanisms.

#### 4.3.3 Cutaneous inflammation

Ginsenoside F2 enriches SCFA-producing gut microbes, markedly elevating fecal and serum propionate levels in atopic dermatitis mice, which positively correlates with the significant expansion of intestinal *P. goldsteinii* (Li et al., 2024a). The augmented propionate suppress the gut-skin inflammatory cascade via the GPR43/NF-κB pathway, thereby alleviating atopic dermatitis symptoms and highlighting the role of the gut microbiota-metabolite-skin axis in systemic anti-inflammatory responses (Li et al., 2024a). Additionally, orally or topically administered Pg-OMVs target inflammatory skin lesions, attenuate immune cell infiltration and alleviate psoriatic pathology. These findings confirm the potential of gut microbiota-derived bioactive components to regulate distal inflammation through the gut-skin axis and establish a novel non-viable microbiota-based delivery strategy for psoriasis therapy (Su et al., 2025). Collectively, these findings suggest that targeting *P. goldsteinii* and its functional components modulates the gut-skin interaction network, offering innovative cross-organ therapeutic strategies for immune-mediated dermatoses.

#### 4.3.4 Infections

Studies indicate that *P. goldsteinii* mitigates infection-associated inflammation through multifaceted mechanisms: its MTS01 strain antagonizes *H. pylori* colonization, suppresses the expression of virulence factors VacA and CagA, and ameliorates infectious gastritis by remodeling the gastroduodenal microbiota structure (Lai et al., 2022a). In influenza A virus infection models, compared with the untreated model group, Xiyanping injection reduces mortality and alleviates alveolar inflammatory damage by enriching *P. goldsteinii* and other beneficial taxa, thus balancing the IL-6/IFN-γ inflammatory-antiviral axis in the lungs (Liu et al., 2025). Collectively, these findings indicate that *P. goldsteinii* mediates cross-pathogen mucosal protection against bacterial and viral infections via a microbiota-immune regulatory network, proposing novel therapeutics targeting host-microbe interactions for anti-infective interventions.

#### 4.3.5 Chronic obstructive pulmonary disease (COPD)

Chronic obstructive pulmonary disease ranks as the third leading cause of death worldwide and a major public health concern. In 2019, there were 212.3 million prevalent cases of COPD globally, resulting in 3.3 million deaths (GBD Chronic Respiratory Disease Collaborators, 2020; Safiri et al., 2022). *P. goldsteinii* significantly mitigates smoking-induced COPD through multi-organ mechanisms: it reduces intestinal inflammation, enhances mitochondrial and ribosomal activity in colonic epithelia, restores host amino acid metabolism, and suppresses pulmonary inflammation (Lai et al., 2022b). Notably, its LPS exhibits anti-inflammatory properties by antagonizing the TLR4 signaling pathway. These collective findings highlight *P. goldsteinii* as a novel therapeutic candidate for COPD, offering dual modulation of gut-lung axis dysfunction and TLR4-driven inflammation (Lai et al., 2022b).

### 4.4 Tumors

Recent studies establish *P. goldsteinii* as a central regulator in CRC continuum care through multistage modulation of the gut-liver immunity and microbial metabolism. Key mechanisms include:

(i) Synergistic restoration of gut microbiota homeostasis with *Ophiocordyceps colitis*-associated tumorigenesis (surpassing *Cordyceps militaris* efficacy), highlighting potential against CRC precursor lesions like IBD (Ji et al., 2021);

(ii) Enhanced anastomosis healing post-CRC surgery via anti-inflammatory activity, countering *Alistipes onderdonkii*- mediated impairment (Hajjar et al., 2023);

(iii) Inhibition of hepatic metastasis by driving KCs expansion, elucidating microbiota-immune cross-organ antitumor mechanisms (Yuan et al., 2022b);

(iv) Sex-biased abundance inversely correlating with pathogenic *A. muciniphila* in male CRC models, identifying gender-specific therapeutic targets (Wang et al., 2023); and

(v) Facilitation of 17 β-estradiol (E2) and anti-PD-L1 synergy by reversing immune checkpoint inhibitor resistance through microbiota remodeling (Song et al., 2023).

Notably, *Schizophyllum commune* polysaccharides indicate dual anti-tumor activity in glioblastoma models by enriching *P. goldsteinii* and upregulating ARHI expression, expanding host-microbe coevolution applications in cancer therapy (Zheng et al., 2024). Collectively, these findings confirm *P. goldsteinii* as a master microbial regulator in CRC management, proposing a microbiota-centric paradigm for precision oncology across prevention, metastasis suppression, and therapeutic optimization.

### 4.5 Allergy

*Parabacteroides goldsteinii* bidirectionally modulates allergic disease progression through a metabolite-immune interplay. In allergy prevention, perinatal goat milk feeding enriches intestinal *P. goldsteinii* in neonatal mice, driving immune maturation of gut-associated lymphoid tissue and suppressing house dust mite-induced Th2 inflammation and reducing airway eosinophilic infiltration (highlighting microbiota-targeted dietary interventions’ potential) (Kao et al., 2020). However, its immunomodulatory effects exhibit context-dependent complexity: penicillin-induced *P. goldsteinii* expansion increases peripheral CD19 + B cell counts but disrupts CD4+/CD8+ T cell ratios, suggesting that microbiota-specific alterations may perturb immune homeostasis (Daniluk et al., 2017). Clinical studies further reveal ancestry-dependent functional divergence–elevated *P. goldsteinii* abundance in Black children with food allergies positively correlates with asthma risk, implicating host-microbe coevolution in allergic susceptibility (Mahdavinia et al., 2023). Supplementation with short-chain fructans (kestose) and inulin selectively enhances *P. goldsteinii* proliferation, potentiating SCFA production to alleviate ovalbumin -induced hypersensitivity via IL-4 inhibition and IL-10 induction (Takahashi et al., 2023). We hypothesize that these effects may be modulated by host genetics (e.g., FXR or GPR43 polymorphisms), habitual diet (fiber intake), and environmental factors (antibiotic exposure), which could account for observed inter-individual variability and warrant future stratified clinical trials. These findings collectively position *P. goldsteinii* as a metabolic orchestrating of allergic regulation, though mechanisms underlying ancestry-specific microbiota-immune crosstalk require systematic dissection through multi-omics approaches to enable clinical translation.

### 4.6 Irritable bowel syndrome (IBS)

*Parabacteroides goldsteinii* mediates pivotal pathophysiological mechanisms role in the IBS (Yu et al., 2021). Red ginseng (RG) intervention significantly ameliorates intestinal hypersensitivity and anxiety-like behaviors in IBS murine models, demonstrating efficacy comparable to first-line clinical therapeutics. Mechanistic studies establish that these benefits require RG-induced enrichment of intestinal *P. goldsteinii*. Specifically, RG promotes *P. goldsteinii* proliferation to suppress pro-inflammatory cytokine (e.g., IL-1β) release while bidirectionally modulating key microbiota-gut-brain axis mediators–as demonstrated by corticosterone downregulation and c-Fos activation–thereby synergistically alleviating visceral pain and central nervous comorbidities (Yu et al., 2021). These findings position *P. goldsteinii* as a potential therapeutic target for IBS while offering a translational framework for neurogastroenterological interventions leveraging microbiota-host crosstalk.

### 4.7 Systemic autoimmune diseases

The regulatory role of *P. goldsteinii* in systemic autoimmune diseases has garnered increasing attention. Studies have indicated that this bacterium and its secreted OMVs suppress NET formation by activating the Cav-1-Nrf2 signaling axis, thereby mitigating inflammatory progression in RA and revealing novel microbiota-immune regulatory mechanisms in autoimmunity (Ye et al., 2025). Further investigations indicate that both *P. goldsteinii* and the medicinal fungal polysaccharides (*Hirsutella sinensis*) can re-establish immune homeostasis, markedly reduce anti-dsDNA autoantibody levels, and alleviate renal and splenic pathological damage in systemic lupus erythematosus (SLE) mouse models (Chang et al., 2024). Collectively, these findings establish *P. goldsteinii* as a cross-disease regulatory hub in autoimmunity, pathogenesis, advancing targeted intervention strategies leveraging microbiota-derived signaling molecules (e.g., OMVs) or microbe-drug.

## 5 Determinants of *P. goldsteinii* abundance: pharmacological and lifestyle modulators

### 5.1 Pharmacological interventions

Pharmacological agents dynamically regulate *P. goldsteinii* abundance and functional activity through direct antimicrobial effects or indirect modulation of host-microbiota crosstalk. Metformin–a first-line type 2 diabetes (T2D) therapy–reprograms BA metabolism by suppressing primary BA (PBA)-producing *Weissella* spp. and reducing secondary BA-converting *Parabacteroides* spp. (Zhang et al., 2023). This dysbiosis downregulates anti-inflammatory genes (e.g., JUND) and inhibits *P. goldsteinii* colonization via FXR signaling suppression, ultimately exacerbating intestinal barrier dysfunction in intolerant patients (Zhang et al., 2023). Similarly, gender-affirming hormone therapy induces sex steroid-driven gut microbiota restructuring, with *P. goldsteinii* abundance fluctuations suggesting sex hormones modulate its ecological niche competition through immune-metabolic axis rewiring (Liwinski et al., 2024). Non-steroidal anti-inflammatory drugs (NSAIDs, e.g., aspirin) exhibit a bidirectional relationship with *P. goldsteinii*: NSAID-induced gut injury reduces *P. goldsteinii* abundance and its protective metabolites (7-keto-LCA), while exogenous supplementation of *P. goldsteinii* or its metabolites reverses epithelial damage by reactivating FXR-mediated repair pathways (Li et al., 2024b). Collectively, pharmacological agents constitute critical modulators of *P. goldsteinii* ecology, offering rationale to optimize therapeutic regimens or develop microbiota-targeted adjuvants.

### 5.2 Dietary and lifestyle modulators

Dietary components and lifestyle patterns govern *P. goldsteinii* colonization through metabolite-mediated microbial interactions, immune regulation, and host circadian rhythm integration. Notably, fermentable fibers, such as inulin increase *P. goldsteinii* abundance and upregulate its outer membrane protein A (OmpA), which subsequently activates IL-22 signaling to stimulate antimicrobial peptide (e.g., Regenerating islet-derived protein 3 gamma/beta; REG3γ/β) secretion (Wang et al., 2024). This establishes a self-reinforcing immune-microbial loop critical for gut homeostasis (Wang et al., 2024). Furthermore, *P. goldsteinii*-derived acetate and butyrate can modulate host circadian gene expression in intestinal epithelial cells by inhibiting histone deacetylases *in vitro* (Fawad et al., 2022). Whether this mechanism synchronizes host-microbe circadian rhythms *in vivo* remains to be directly demonstrated for *P. goldsteinii*. Critically, individuals with balanced metabolic profiles exhibit higher *P. goldsteinii* abundance than dysbiotjc counterparts, suggesting sustained adherence to fiber-rich diets, prebiotics, and regular exercise maintains microbial diversity (e.g., SCFA levels) and metabolic homeostasis (e.g., glycogen reserves). These practices mitigate anemia risk and comorbid disorders (Liang et al., 2024). Collectively, these mechanistic insights position dietary and lifestyle interventions as precision tools for *P. goldsteinii*-centric microbiota engineering.

## 6 Therapeutic applications and technical limitations

### 6.1 Probiotic development and prebiotic synergy

*Parabacteroides goldsteinii* exhibits therapeutic potential through multiple mechanisms (Figure 1): immunomodulation via SCFA-mediated suppression of NF-κB signaling, reducing pro-inflammatory cytokines (e.g., TNF-α, IL-6) while enhancing regulatory T cell (Treg) differentiation (Su et al., 2025); metabolic regulation through improved glucose/lipid homeostasis and insulin sensitivity (Wu et al., 2019; Cai et al., 2020); and intestinal barrier reinforcement by upregulating tight junction proteins (ZO-1, occludin). However, its obligate anaerobic physiology impedes industrial-scale production, necessitating specialized culturing systems (e.g., anaerobic chambers, reduced media) that incur high costs and instability. Innovative solutions include: (i) Building on research for other obligate anaerobes (e.g., *Bacteroides* spp. and *Clostridium* spp.), develop co-culture strategies pairing *P. goldsteinii* with facultative anaerobes (e.g., engineered *E. coli*) to deplete oxygen and create anaerobic micro-niches (Kim et al., 2022; Brown et al., 2021, 2023); (ii) Cryoprotectant formulations (trehalose, skim milk) to enhance viability during lyophilization; (iii) Encapsulation technologies (e.g., alginate-chitosan matrices) for gastric acid resistance and targeted intestinal delivery (Han et al., 2020); and (iv) Standardized strain screening for anti-inflammatory activity and colonization efficiency, combined with synbiotic formulations (e.g., arabinoxylan) to enable clinical translation (Zhou et al., 2024).

### 6.2 Fecal microbiota transplantation (FMT): dose-response dynamics

While FMT restores *P. goldsteinii* abundance in conditions like IBD and obesity (Wu et al., 2019), its therapeutic contribution remains obscured by donor microbiota complexity. Advanced multi-omics approaches (metagenomics, metabolomics) are required to deconvolute *P. goldsteinii*-specific effects from polymicrobial interactions. Metagenomics and metabolomics serve as complementary analytical frameworks in FMT research, enabling mechanistic dissection of FMT efficacy through structural and functional dimensions (Yu et al., 2023). Specifically: 16S rRNA gene sequencing or shotgun metagenomics quantitatively profiles taxonomic composition, α-diversity shifts, and donor-strain engraftment in recipients pre- and post-FMT. These methods track, for instance, *P. goldsteinii* colonization efficiency. Metagenomic functional annotation identifies microbial metabolic pathways, determining whether FMT restores host metabolic homeostasis via specific gene transfers (e.g., buk encoding butyrate kinase). High-coverage sequencing resolves single-nucleotide polymorphisms (SNPs) between donor and recipient strains, thereby identifying competitively dominant taxa–such as antioxidant-enriched *A. muciniphila* strains–that achieve successful engraftment. Predictive models integrating host factors (baseline microbiota, immunogenetics) may identify FMT-responsive subpopulations. *Ex vivo* intestinal organoid systems enable dose-dependent analysis of *P. goldsteinii*-mediated anti-inflammatory effects, thereby guiding personalized FMT protocols.

### 6.3 Synthetic biology approaches

CRISPR-based engineering of *P. goldsteinii* may yield smart therapeutic strains: (i) Context-responsive systems: ROS-inducible IL-10 expression for targeted anti-inflammatory therapy in colitis models (Skrypnyk et al., 2024); (ii) Precision delivery platforms: Engineered strains secreting metabolites (e.g., 7-keto-LCA) to repair NSAID-induced epithelial damage.

Current limitations include the lack of genetic tools adapted for strict anaerobes, necessitating development of anaerobic-compatible CRISPR systems and transformation protocols. Key translational barriers: (i) Technical hurdles in large-scale cultivation; (ii) Incomplete understanding of dose-effect relationships; (iii) Paucity of strain-specific genetic toolkits. Overcoming these requires interdisciplinary collaboration to bridge microbial ecology, bioengineering, and clinical pharmacology.

## 7 Conclusion and perspectives

The gut commensal *P. goldsteinii* exhibits broad therapeutic potential against metabolic, autoimmune, neurological and neoplastic disorders by engaging immunomodulatory, metabolic, barrier-reinforcing and gut–organ axis pathways. Yet clinical translation is stalled by several evidence gaps. Most studies rely on small-sample murine models (e.g., diet-induced obesity, chemically triggered colitis) or *in vitro* organoids that inadequately capture human microbiome complexity and disease heterogeneity. Murine TLR4 or FXR pathways may not mirror human physiology, and complex interventions (herbal extracts, synbiotics) obscure strain-specific effects.

Crucially, no phase I/II/III trials of *P. goldsteinii* monotherapy exist. Human data are purely correlative: reduced abundance is reported in IBD, whereas elevated levels associate with asthma in food-allergic children–underscoring context dependency that demands human validation. Live-biotherapeutic development is further constrained by strict anaerobiosis, uncharacterized long-term safety in immunocompromised hosts, and undefined potency, dosage or combination protocols.

Future priorities include (i) proof-of-concept trials in well-defined cohorts (e.g., *P. goldsteinii*-deficient IBD or MASLD patients); (ii) gnotobiotic, knockout and single-cell omics approaches to isolate strain-specific mechanisms; (iii) engineering of aerotolerant variants or non-viable derivatives (OMVs, 7-keto-LCA) for targeted delivery; and (iv) global consortia to standardize strain biobanking, harmonize endpoints and guide regulation. Until robust human data and scalable delivery solutions are secured, *P. goldsteinii* remains a promising but pre-clinical candidate for precision microbiome therapy.
